# Pre-Processing Effect on the Accuracy of Event-Based Activity Segmentation and Classification through Inertial Sensors

**DOI:** 10.3390/s150923095

**Published:** 2015-09-11

**Authors:** Benish Fida, Ivan Bernabucci, Daniele Bibbo, Silvia Conforto, Maurizio Schmid

**Affiliations:** Department of Engineering, University of Roma Tre, Via Vito Volterra, 62, Rome 00146, Italy; E-Mails: ivan.bernabucci@uniroma3.it (I.B.); daniele.bibbo@uniroma3.it (D.B.); silvia.conforto@uniroma3.it (S.C.); Maurizio.schmid@uniroma3.it (M.S.)

**Keywords:** inertial measurement unit, gait event detection, dynamic segmentation, pre-processing, physical activity, classification

## Abstract

Inertial sensors are increasingly being used to recognize and classify physical activities in a variety of applications. For monitoring and fitness applications, it is crucial to develop methods able to segment each activity cycle, e.g., a gait cycle, so that the successive classification step may be more accurate. To increase detection accuracy, pre-processing is often used, with a concurrent increase in computational cost. In this paper, the effect of pre-processing operations on the detection and classification of locomotion activities was investigated, to check whether the presence of pre-processing significantly contributes to an increase in accuracy. The pre-processing stages evaluated in this study were inclination correction and de-noising. Level walking, step ascending, descending and running were monitored by using a shank-mounted inertial sensor. Raw and filtered segments, obtained from a modified version of a rule-based gait detection algorithm optimized for sequential processing, were processed to extract time and frequency-based features for physical activity classification through a support vector machine classifier. The proposed method accurately detected >99% gait cycles from raw data and produced >98% accuracy on these segmented gait cycles. Pre-processing did not substantially increase classification accuracy, thus highlighting the possibility of reducing the amount of pre-processing for real-time applications.

## 1. Introduction

Automatic recognition of locomotor activities from wearable sensors is a crucial task in many health-related applications, especially in the area of assisted living and healthcare. In terms of technology, accelerometers and gyroscopes have received the highest attention in this area, when long-term and personal monitoring is sought: when combined together in an inertial sensor unit, they can be used to automatically and robustly segment the different phases of an activity cycle (generally, a gait cycle) and possibly determine information associated with the quality of each cycle. The temporal accuracy of segmentation techniques can also have an impact on the reliability of the subsequent steps (classification and qualification). Different segmentation methods have been used in the past: most common among them are those using a fixed window size and those based on the identification of gait events.

In the first class of techniques, the signal is divided into consecutive windows of fixed length, which, in the case of physical activities (level walking and stair walking) lie in the range of 1–10 s [1,2,3,4,5,6,7,8]. One limitation of this approach is that if an activity lasts for shorter or longer time periods than the pre-defined chosen window length, the subsequent classification might be affected.

When using a gait event-based segmentation, instead, windows are segmented based on the identification of one or more gait events, such as the foot strike or the foot-off. For instance, gyroscopes placed at the shank level have been proven to be acceptably accurate in healthy gait walking up and down an incline [9] and in pathological [10,11] and in healthy gait when walking on level ground [10,12]. Gyroscopes placed on the foot and on the shank have been used for locomotion pattern classification, including descending and ascending stairs [13,14,15]. Event-based segmentation techniques can use a small fixed-length window to identify the events within one gait cycle (initial contact, flat foot or foot-off) [16,17,18,19]. Chen *et al.* extracted all peaks (mid-swing) from the accelerometer anterior-posterior component and used the center of two consecutive peaks to identify the flat-foot event within a search window that is a part of the estimated gait cycle [13]. In [20], each foot-off event was detected based on the local minimum search from the expected foot-off point to zero-crossing of the next swing phase.

When dealing with a physical activity recognition problem, it has been argued that the de-noising of the inertial sensor data is necessary in order to be able both to extract the relevant information [6,21,22,23] and to identify the gait events from smooth signals [9,13,15,17,24,25]. De-noising can be considered as the combination of the pre-processing steps that are done to minimize the effect of noise (e.g., by filtering) and sensor misplacements (e.g., by considering a reference position for the sensor). For this second source of error, in previous works, it has been argued that the correction of acceleration and angular velocity is necessary due to the inclination correction of the sensor and the effect of the gravity on the signal [24,26]. Table 1 summarizes the differences in pre-processing and segmentation when inertial units are used to recognize physical activities. To the authors’ knowledge, no study has evaluated yet the role of these pre-processing steps on the classification accuracy of the physical activities.

**Table 1:** Studies on different pre-processing approaches.

Study/Sensors	Sensors	Activities	Segmentation/Filtering	Classification Accuracy/Gait Event Detection
Mantyjarvi *et al.* [27]	2 accelerometers	SA, SD, WK, Other	Fixed size (2 s)/filtered	83%–90%/n.a.
Maurer *et al.* [28]	6 accelerometers	RUN, SA, SD, Sit, Std, WK	Fixed size (0.5 s)	87%/n.a.
Lovell *et al.* [29]	1 accelerometer	WK, SA, SD	Fixed size (2.5 s)	92%/n.a.
Lau *et al.* [14]	2 IMUs	WK, SA, SD, SW	Event-based/filtered	85%–100%/n.a.
Chen *et al.* [13]	1 IMU	WK, SA, SD	Event-based/filtered	92%–95%/n.a.
Wang *et al.* [30]	1 smartphone	WK, J, SA, SD	Fixed size (0.5 s, 0.8 s)	93.3%/n.a.
Panahandeh *et al.* [18]	1 IMU	RUN, SA, SD, Std, WK	Event-based/filtered	95%/n.a.
Fraccaro *et al.* [24]	1 accelerometer, 1 gyroscope	WK	Event-based/filtered	n.a./92.5%
Formento *et al.* [16]	1 gyroscope	SA, SD	Event-based/filtered	n.a./93%–95%
Ngo *et al.* [19]	3 IMUs	WK, SA, SD, SW	Event-based/filtered	94%
Chen *et al.* [17]	2 IMUs and foot pressure	WK, SA, SD, SW	Event-based/raw	n.a./90%–100%

IMU, accelerometers and gyroscopes. J: jogging, RUN: running, SA: stair ascending, SD: stair descending, Sit: sitting, Std: standing, SW: slope walking, WK: level walking.

This study thus analyzes and compares the classification accuracy obtained through an event-based dynamic segmentation on different pre-processing operations. In particular, since the goal of this paper is to assist the researcher in building real-time applications, the monitored pre-processing operations will be considered, taking into account the computational complexity associated with their implementation. The main contributions of the paper are thus:

- to investigate whether, and to what extent, de-noising and inclination correction pre-processing has an effect on the segmentation of activities and on the subsequent classification accuracy;
- in order to do so, we chose to present a modification to a standard gait segmentation criterion in such a way that no window is used to detect the events of the physical activities. In this way, we were able to also evaluate the effect of a dynamic event-based segmentation on the ability to classify human physical activities (that were not limited to level walking, but included stair negotiation).

The rest of the paper is organized as follows. We describe data collection in Section 2 and data pre-processing in Section 3. Results are described in Section 4, while Section 5 draws the conclusions and future work.

## 2. Data Collection

In order to evaluate the segmentation accuracy across different datasets, we used two different datasets. Details are reported in the next two subsections.

### 2.1. First Dataset

Nine healthy adults (29 ± 5 years) were recruited for the first dataset. Participants were equipped with an inertial measurement unit that included an ADXL345 tri-axial accelerometer (range *±*39.9 m/s^2^) and an ITG3200 tri-axial gyroscope (±2000 rad/s), fixed on the shank (lateral position) of the dominant leg. The accelerometer x-axis was positioned in the anterior-posterior direction, the y-axis in the inferior-superior direction and the z-axis in the lateral-medial direction. The gyroscope rotations were defined as follows: x-axis (coronal plane); y-axis (transverse plane); z-axis (sagittal plane).

Experiments were carried out in the university building, except running, which was performed outside the campus building. All participants were asked to carry out activities at their self-selected speed and had to walk on a predefined route. During the first 5 s of the experiment, the subjects stood still in an upright position to initialize the offset; then, they followed a route, including a walking path of 50 m, opening and closing a door, stairs ascent (SA, staircase of 46 steps), a few walking steps, opening and closing a door, running (outside the building along the path of about 150 m), opening and closing a door, stairs descent (SD), opening and closing a door, and walking. At the end/start of each activity, subjects stood still for few seconds in order to label the data correctly. Data were collected at a sampling rate of 100 Hz. Labeling was done by visual inspection by one experimenter.

### 2.2. Second Dataset

**Figure 1:**
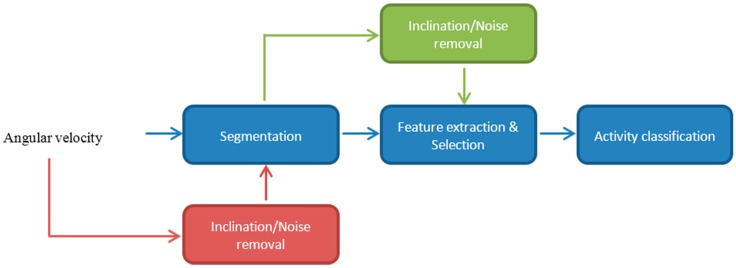
Workflow of the activity recognition chain.

In the second dataset, data were recorded from seven healthy adults with the same sensor settings. In this dataset, the physical activities that were performed by the participants were walking, stairs ascending and stairs descending. In this second dataset, thus, no running activity was performed. Figure 1 shows the flowchart of the activity recognition process.

## 3. Pre-Processing

The work presented here focuses on the effect of the most common pre-processing steps used when gathering inertial sensor data for activity monitoring, *i.e.*, inclination correction and filtering.

### 3.1. Inclination Correction

Due to the nature of the monitored physical activities, the sensor attached to the body is prone to move with respect to the body segment, thus producing an unintended bias in data recording. To minimize the influence of sensor inclination, each data channel value was removed from the average value obtained when standing still for 5 s before starting the activity path.

### 3.2. Signal Filtering

To remove the noise from the signal, an online filter was implemented to smooth the current sample by applying the following Equation (1):

(1) $$y_{n}=\;b_{0}x_{n}+b_{1}x_{n-1}+\ldots +\;b_{M}x_{n-M}-\;a_{1}y_{n-1}-\ldots -\;a_{N}y_{n-N}\;=\;\overset{M}{\underset{k=0}{\sum }}b_{k}x_{n-k}-\;\overset{N}{\underset{k=1}{\sum }}a_{k}y_{n-k}$$

where *y* is the filtered output of the input x, and b and a are the coefficients of the first order low pass filter that were computed by considering a cut-off frequency of 10 Hz, as a cut-off frequency of 10 Hz is motivated by the previous works [13,14]. We have applied different orders of low-pass filtering (*i.e*., choosing different values for N and M) and chose N = M = 2, as this configuration offers pretty low complexity for the implementation, still giving fair values in terms of selectivity (−15 dB at twice the corner frequency, set at 10 Hz).

### 3.3. Dynamic Segmentation (Gait Cycle Detection)

Previous studies have encouraged the use of a shank-mounted gyroscope to detect the gait events [10,14,15,16]. The angular velocity in the sagittal plane has a distinct pattern for locomotor activities, *i.e*., a positive peak during the swing phase, followed by the stance phase. Angular velocity in the sagittal plane g_z_ was used for the gait cycle detection, based on the identification of the foot-off events. To achieve this task, local minima and maxima were updated, and the foot-off event was identified when specific conditions were met, as will be shown in the following.

The algorithm starts to find two successive zero-crossings (a negative zero-cross followed by a positive zero-cross) that are hypothesized as characteristic of the swing phase: the swing phase is thus segmented if the maximum value of the angular velocity in that direction is greater than 1.8 rad/s; under this circumstance, its location is hypothesized as the mid-swing position. Once the swing phase is identified, the algorithm starts searching for the following minimum value, as it corresponds to the foot strike event. Once the foot strike event is found (and located at t*_fs_*), the algorithm searches for the local maximum t*_max_* and local minimum t*_min_* and then verifies the following condition:

(2) $$t_{min}-t_{max}\geq 60\;\text{ms}\;\&\&\;g_{z}\left(t_{min}\right)\leq 1.4\;\text{rad}/\text{s }\&\&\;t_{max}-t_{fs}\geq 70\;\text{ms}$$

If the equation is satisfied, t*_min_* is saved as the estimated location of the foot-off event. If this condition is not satisfied within 1.3 s (as may happen in the case of transitions or when the person is resting), then the algorithm discards the current saved swing phase and starts the search for the next swing phase. Each activity cycle, which represents one segmented window over which features will be extracted, is thus defined by using two consecutive foot-off events.

**Figure 2:**
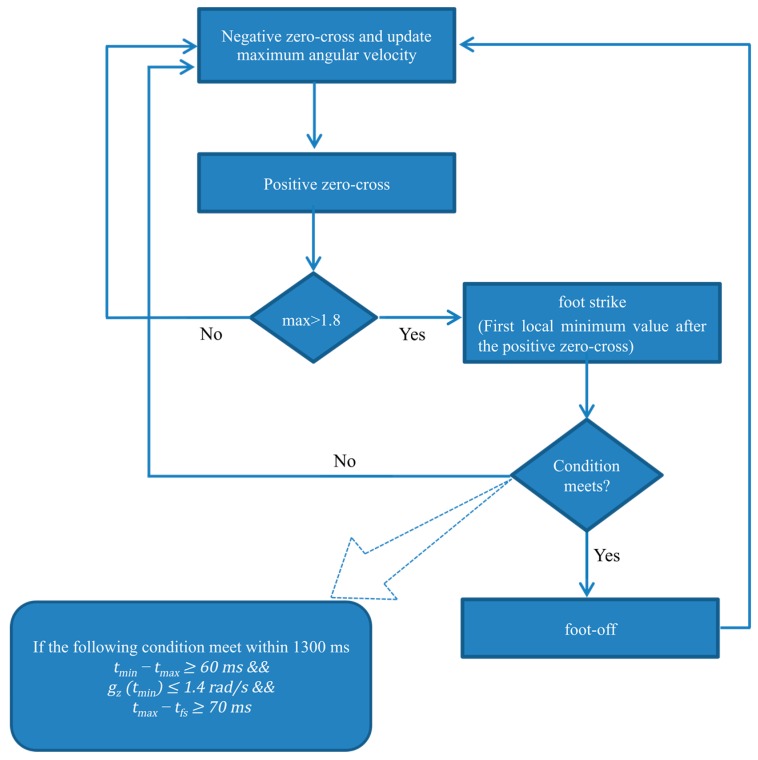
Signal segmentation algorithm flow diagram.

**Figure 3:**
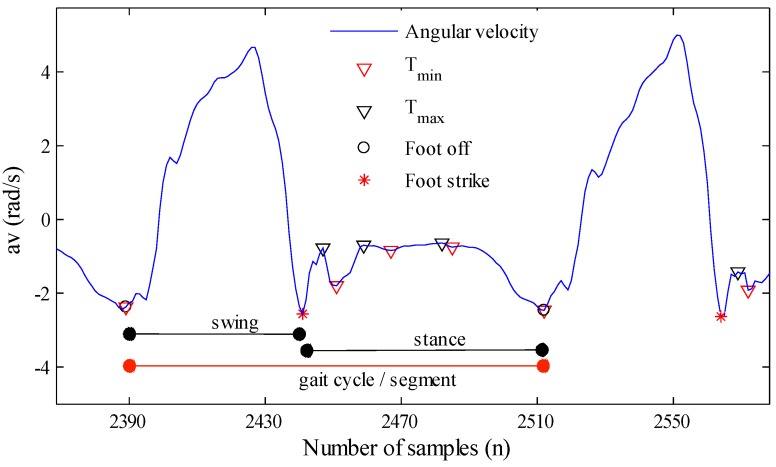
Segmentation algorithm detection for a walking step, where black and red triangles are t*_max_* and t*_min_*, red asterisks are foot strike and black circles are foot-off events.

Segmentation was performed on the raw, inclined and filtered data separately. The flow diagram of the segmentation algorithm and its outcome are presented in Figure 2 and Figure 3, respectively.

### 3.4. Feature Extraction and Selection

Segments identified with the above-mentioned rules were taken as the reference for all of the remaining inertial data. For each segment, a set of time and frequency domain features (mean value, median value, skewness, kurtosis, standard deviation, correlation, interquartile range, energy, first five FFT coefficients) that are used in the literature for the activity recognition problem were derived from each axis and magnitude of the accelerometer and gyroscope signal [1,22].

The signal magnitude is considered as orientation independent and useful in solving the sensor orientation inconsistency problem; it is calculated by applying Equation (3):

(3) $$a_{mag}=\sqrt{a_{x}^{2}+\;a_{y}^{2}+\;a_{z}^{2}}\;,\;g_{mag}=\sqrt{g_{x}^{2}+g_{y}^{2}+g_{z}^{2}}$$

**Figure 4:**
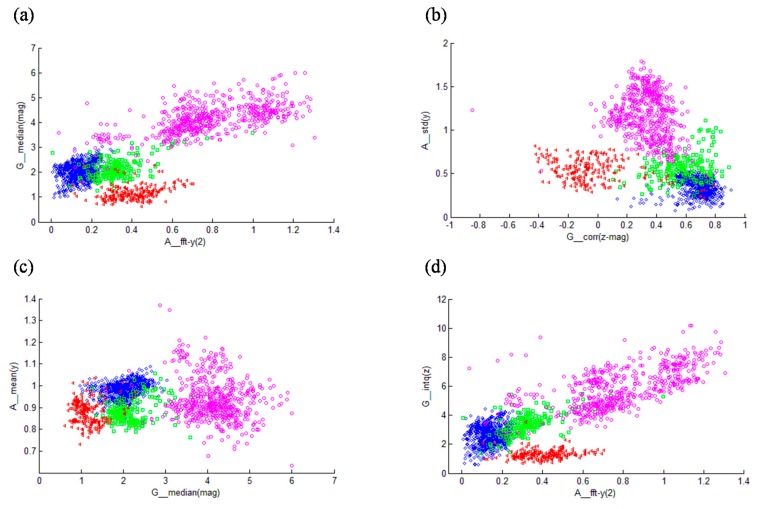
An example of activity clusters’ distribution over features selected by SVM, where pink, red, green and blue colors represent running, SA, SD and walking activities, respectively. (**a**–**d**) refer to different combinations of feature pairs.

A total number of 152 features were thus extracted. Feature selection was done through the SVM classifier, where attributes were ranked by the square of the weight assigned by the SVM [31], and the first 20 features were selected based on the experiments to classify the activities. The selected features are listed in Table 2 and Figure 4 shows the activity cluster representation for the extracted features.

**Table 2:** SVM selected features.

Features	Time Domain	Frequency Domain
Mean	a_x_, a_y_	
Median	a_y_, g_z_, g_mag_	
Skewness	a_z_	g_z_
Standard deviation	a_x_, a_y_	
Correlation	g_z_, g_mag_	
Interquartile	g_z_	
Energy		a_x_
FFT coefficients		a_x_ (2nd), a_y_ (1st, 2nd, 3rd, 5th), a_mag_ (1st, 2nd), g_z_ (3rd)

The first five FFT coefficients (as calculated over each segment) were used, as these contain the main frequency components (up to 5 Hz). The features selected by the algorithm are also used in Shoaib *et al.* [1,7] and are considered useful for running on smartphones, as they have very low or medium computational and storage complexity.

### 3.5. Classification

We tested the recognition performance of the support vector machine (SVM) classifier on the selected features. Some studies pointed out the positive performance of the SVM in the activity recognition problem [32,33]. In SVM, the multi-class problem was solved by using pairwise classification (1 *vs*. 1 [34]). Different kernel methods with varying complexity parameters were tested, and a polynomial kernel with a complexity value of one performed best on the problem. Classification was performed in WEKA Experimenter (University of Waikato) [35].

The leave-one-subject-out cross-validation criterion was used on the first dataset to evaluate the performance of the classifier: data of one subject were used for the testing, while the data of the remaining subjects were used for training; this process was repeated for all subjects. In the case of the second dataset, the classifier was trained on the overall data of the first dataset, and testing was performed on the second one. The final result represents the average accuracy over all subjects.

## 4. Results and Discussion

### 4.1. Signal Segmentation

Signal segmentation was carried out on six different configurations of pre-processing to be tested: segmentation performed on raw signals (no further processing); segmentation performed on signals corrected for inclination (no further processing); segmentation performed on raw signal (then filtering applied on segmented windows); segmentation performed on signals corrected for inclination (then filtering applied on segmented windows); segmentation performed on filtered signals (no correction for inclination); segmentation performed on filtered signals (corrected for inclination).

The position of the inclination correction in the processing step does not have an effect on the segmentation quality, since the criteria based on absolute values for the angular velocity in the processing steps for segmentation are not affected by inclination correction.

**Figure 5:**
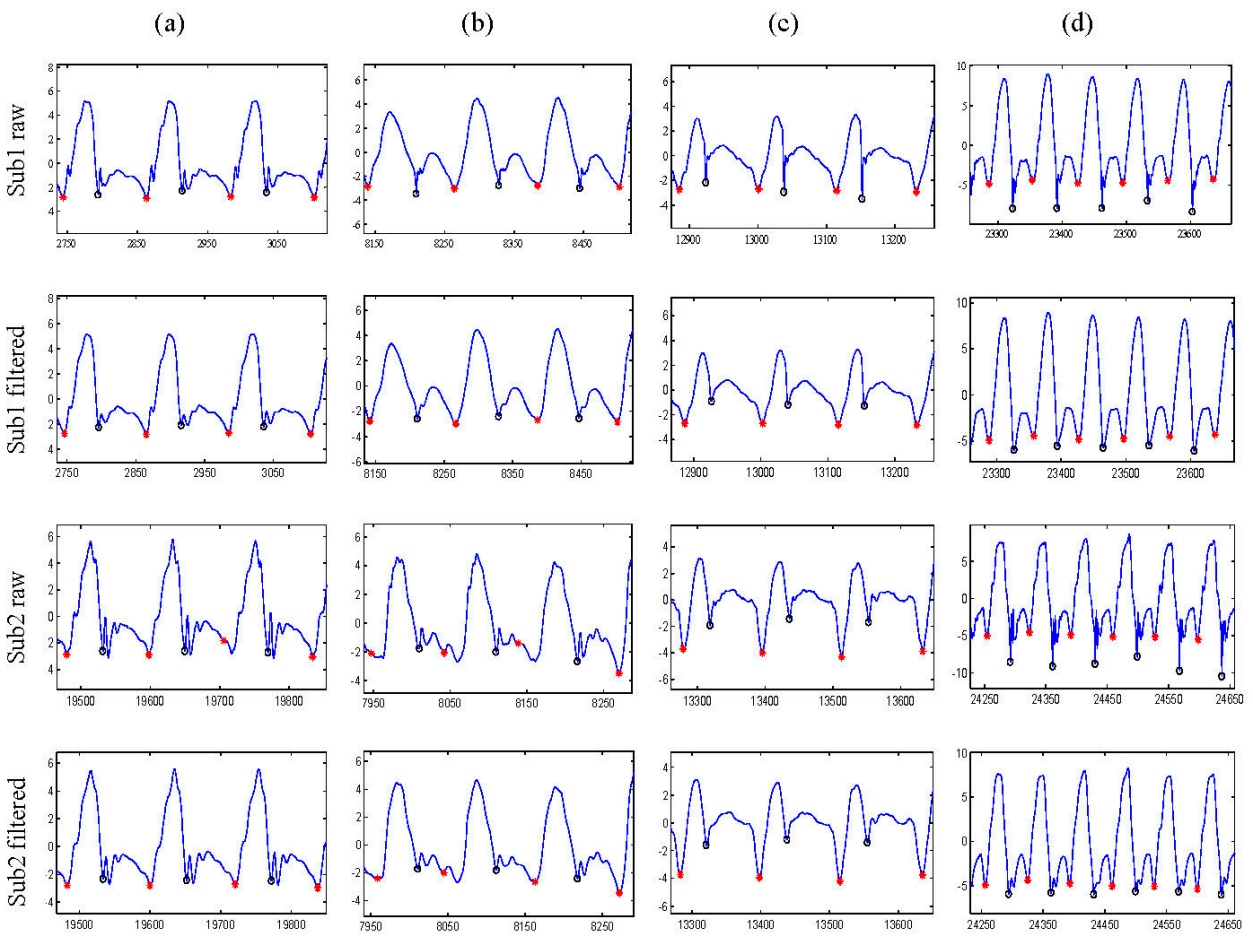
Signal segmentation based on gait events; zero circles are foot strike events, and red asterisks are foot-off events: (**a**) walking; (**b**) stairs descending; (**c**) stairs ascending; (**d**) running.

Conversely, some earlier detections of foot-off events appeared in raw signals in some subjects, as compared to filtered signals. These differences are sometimes present in walking and stairs descending activities, as shown in the columns of Figure 5a,b.

### 4.2. Classification Results

**Figure 6:**
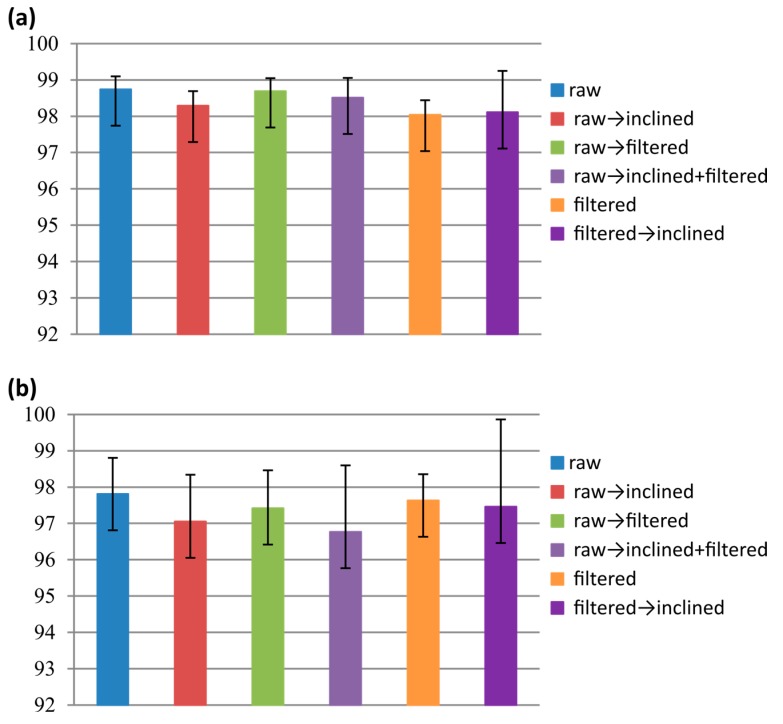
Average classification accuracy over; (**a**) first dataset; (**b**) second dataset.

Segments obtained from the six different pre-processing settings were fed to SVM to classify the ongoing activity. Figure 6 illustrates the average classification accuracy on both datasets.

Classification performance of the event-based segmentation on the first dataset is pretty high (average accuracy >98% for all settings), with less variation among the pre-processing settings. In the second dataset, accuracy obtained with different settings has shown a higher (still not relevant) variation: as expected, average performance decreased for all configurations. In both datasets, classification obtained with the raw signal resulted in being slightly higher than all of the other configurations. Standard deviation (obtained by considering the different runs of the leave-one-subject-out approach) bars show that variation in performance among subjects’ performance increases when inclination is removed from the signal, as compared to the other configurations. To compare the results with previous studies, the performance of each activity on raw data is shown in Table 3.

The main idea of this study is the analysis of the gait inertial signal. There are a number of studies confined to the signal segmentation and activity recognition, but all of them are following the trend of the signal de-noising and window-based segmentation. However, these preprocessing steps may increase the system time and space complexity, when the problem is associated with real-time applications.

**Table 3:** Activity performance evaluation over raw data.

Activities	Confusion Matrix				Performance Measures (%)		
	WK	SD	SA	RUN	Specificity	Sensitivity	Step Detection
WK	**99.3**	0.3	0.3	0.1	98.03	99.3	99.75 (1244/1247)
SD	3.4	**96.6**	0	0	98.53	96.6	98.9 (369/373)
SA	2.8	0	**97.2**	0	98.6	97.2	99.2 (372/376)
RUN	0.4	0.2	0.1	**99.3**	99.82	99.3	100 (570/570)

WK: walking, SD: stairs descending, SA: stairs ascending, RUN: running.

### 4.3. The Role of Segmentation

The signal segmentation algorithm proposed in this study is based on heuristic rules and sequentially evaluates each sample. Furthermore, it has been reported that a rule-based algorithm performed nine-times faster than wavelets analysis-based algorithms [9,11], which represents an advantage for on-line systems. Identification of the gait events was performed by considering timely detection, without using any window for backward or forward search for events. In our study, differences in the detection of the gait cycles from the raw gyroscope (z-axis) signal and from other pre-processing settings are not very significant, since all configurations reached an average detection accuracy >99%, which is in accordance with other studies. Where Formento *et al.* [16] reported 95% event detection in stairs walking, Faracarro *et al.* [24] achieved 92.5% walking event detection, and Catalfamo *et al.* [9] achieved 98% gait event detection accuracy during ground walking and slope waking. In the mentioned studies, event detection was performed on the filtered signal, and a window size of 80 ms to 250 ms was used to detect the foot-off event, and an additional 50 to 135 ms of event location difference was found.

### 4.4. The Role of De-Noising on Classification

Foot-off events detected by the segmentation algorithm were taken as the reference point for the start and end of each activity cycle over which the features were extracted. Referring to the classification accuracy among pre-processing settings, the performance obtained with the raw signal is consistent in both datasets, but not significantly different from other settings. In this study, the feature set that is used to classify the activities is optimal; the information retrieved by these features was not affected by the presence or absence of the noise in the signal. The classification accuracy obtained from raw and filtered data is >98%, which is higher than the previous studies, where Chen *et al.* [13] achieved 94% accuracy, Panahandeh *et al.* [18] 95%, Coley *et al.* [15] 92.5% to classify between stairs ascent and other (walking and stairs descent), Ngo *et al.* [19] 94% and Chen *et al.* [17] reported a 10.78% error with single stance, a 3.42% error with double stance, and a 5.6% error when looking at swing phase-based recognition. All of these studies carried out the classification process on de-noised and event-based segmented data, except Ngo *et al.* [19].

### 4.5. Final Considerations

Different gait event detection algorithms have been used in the literature; rule-based segmentation [9,11,16], hidden Markov model [18] and wavelet analysis [12,15]. All of these studies validated their results on de-noised accelerometer/gyroscope signals and considered small windows for event detection. The performance of these algorithms shows sufficient reliability from 92% to 99% to detect gait events, but when they are applied to the classification of the activities from those segmented events, the highest achieved accuracy is 95%; while the segmentation detections achieved by our algorithm on raw gyroscope signal are in accordance with these studies, and the classification results are higher (>98%) than previous studies.

Our findings show that the pre-processing operators, inclination removal and signal de-noising, have no significant impact on the segmentation and average classification of the physical activities. However, there is a small difference in the earlier detection of the gait events among raw and filtered signals, but the results show that this difference does not affect the classification performance. One might consider the use of raw inertial sensor data for the dynamic segmentation and classification of the daily locomotion activities from the shank-mounted inertial sensor, as the use of noise removal steps has no significant effect on the segmentation and classification of the activities, while it increases the complexity of the system for online applications: if the filtering used is composed of just two taps, at least 2 × 10 ms will be needed as the waiting time, to have the preceding samples available for the processing.

## 5. Conclusions

We evaluated the activity recognition performance on two datasets with different pre-processing settings from a shank-mounted sensor. Four different physical activities (walking, stairs ascending/descending and running) were targeted in our dataset, as these activities are considered as common daily living activities and are associated with gait event detection. The purpose of evaluating the results on different settings is to assist the real-time application in time and space complexity.

The research and associated experiments presented here showed that the proposed system is highly accurate in gait cycle identification >99% and physical activity classification >98% from these gait cycles on raw data. The algorithm can be easily implemented on the smartphone for the real-time application of physical activity classification, as it reduces the computational and storage complexity associated with pre-processing. It is possible to identify the gait cycle based on simple rules from the raw signal. Additionally, features extracted from the signal segments are also considered as having very low or medium computational and storage complexity for real-time applications.

## 6. Limitations and Future Work

The main limitation of this study is that the data collection cannot be fully considered as a realistic scenario, as we only considered the door opening and closing activity while walking (thus leaving out a number of activities that can be done while walking or standing); this activity did not affect event detection. Other household activities, like computer work, dish washing or object moving, could be included to further investigate the performance of the detection algorithm. Future work needs to be considered to include the strides from elderly group or people with neurological/orthopedic disorders, to check whether the algorithm is able to perform well also in these more challenging conditions.
